# Augmented pathogen detection in brain abscess using metagenomic next-generation sequencing: a retrospective cohort study

**DOI:** 10.1128/spectrum.00325-24

**Published:** 2024-09-12

**Authors:** Xuyang Wang, Xiaoxiao Guo, Hong Liu, Bei Wang, Jing Wu, Shengsen Chen, Wenhong Zhang, Xinyun Zhang, Xinyu Wang

**Affiliations:** 1 Department of Infectious Diseases, Shanghai Key Laboratory of Infectious Diseases and Biosafety Emergency Response, National Medical Center for Infectious Diseases, Huashan Hospital, Shanghai Medical College, Fudan University, Shanghai, China; 2 Department of Laboratory Medicine, Huashan Hospital, Shanghai Medical College, Fudan University, Shanghai, China; 3 Department of Endoscopy, Cancer Hospital of the University of Chinese Academy of Sciences (Zhejiang Cancer Hospital), Institute of Cancer and Basic Medicine (IBMC), Chinese Academy of Sciences, Hangzhou, China; 4 National Clinical Research Center for Aging and Medicine, Huashan Hospital, Fudan University, Shanghai, China; 5 Key Laboratory of Medical Molecular Virology (MOE/MOH) Shanghai Medical College, Fudan University, Shanghai, China; MultiCare Health System, Tacoma, Washington, USA

**Keywords:** brain abscess, cerebral abscess, next-generation sequencing, molecular technique, anaerobic bacteria

## Abstract

Brain abscess is a severe infection characterized by the accumulation of pus within the brain parenchyma. Accurate identification of the causative pathogens is crucial for effective treatment and improved patient outcomes. This 10-year retrospective, single-center study aimed to compare the detection performance of conventional culture methods and metagenomic next-generation sequencing (mNGS) in brain abscess. We reviewed 612 patients diagnosed with brain abscess and identified 174 cases with confirmed etiology. The median age was 52 years, with 69.5% males. Culture tests predominately identified gram-positive bacteria, particularly *Streptococcus* spp. Gram-negative bacteria, including *Klebsiella* spp., were also detected. However, mNGS revealed a more diverse pathogen spectrum, focusing on anaerobes (e.g., *Fusobacterium* spp., *Parvimonas* spp., *Porphyromonas* spp.*, Prevotella* spp., and *Tannerella* spp.). mNGS exhibited significantly higher overall pathogen-positive rates in pus samples (85.0% vs 50.0%, *P* = 0.0181) and CSF samples (84.2% vs 7.9%, *P* < 0.0001) compared to culture. Furthermore, the detection rates for anaerobes displayed a notable disparity, with mNGS yielding significantly higher positive detections in both pus samples (50.0% vs 10%, *P* = 0.0058) and CSF samples (18.4% vs 0%, *P* = 0.0115) when compared to culture methods. The assistance of mNGS in pathogen detection, particularly anaerobes in brain abscess, was evident in our findings. mNGS demonstrated the ability to identify rare and fastidious pathogens, even in culture-negative cases. These results emphasize the clinical value of mNGS as a supplement for brain abscess, enabling more comprehensive and accurate pathogen identification.

**IMPORTANCE:**

The accurate identification of pathogens causing brain abscess is crucial for effective treatment and improved patient outcomes. In this 10-year retrospective study, the detection performance of conventional culture methods and metagenomic next-generation sequencing (mNGS) was compared. The study analyzed 612 patients with brain abscess and confirmed etiology in 174 cases. The results showed that culture tests predominantly identified gram-positive bacteria, while mNGS unveiled a broader diverse pathogen spectrum, particularly anaerobes. The mNGS method exhibited significantly higher overall rates of pathogen positivity both in pus and cerebrospinal fluid (CSF) samples, surpassing the culture methods. Notably, mNGS detected a significantly higher number of anaerobes in both pus and CSF samples compared to culture methods. These findings underscore the clinical value of mNGS as a supplement for brain abscess diagnosis, enabling more comprehensive and accurate pathogen identification, particularly for rare and fastidious pathogens that evade detection by conventional culture methods.

## INTRODUCTION

Brain abscess is a critical pathological condition characterized by the localized accumulation of pus within the brain parenchyma (1, 2). It typically arises from infections that spread hematogenously or through contiguous tissues, originating from primary sources such as sinusitis, otitis media, mastoiditis, dental infections, or lung abscesses (3). Additionally, brain abscess can manifest as a complication of neurosurgical procedures or penetrating head trauma (4).

The diagnosis of brain abscess poses a significant challenge due to its variable clinical presentation and the difficulty of obtaining pus material for microbiological identification. Surgical and traditional culture procedures considerably lengthen the diagnostic process and hamper antimicrobial treatments. While oral cavity bacteria, containing many anaerobes, are the most frequent causative pathogens, the positive culture rate of anaerobic bacteria is highly variable (2, 5, 6). Culture-based methods suffer from limitations including low sensitivity, prolonged turnaround time, and the requirement for viable organisms (7). These drawbacks are particularly evident when identifying anaerobic bacteria with complex growth requirements that challenge standard laboratory culturing techniques (8). Although computed tomography (CT) and magnetic resonance imaging (MRI) are crucial for the diagnosis of brain abscess, their contribution to pathogens differentiation is notably limited (9).

Identifying the causative pathogen is crucial for effectively managing brain abscess. Delayed or missed diagnoses can result in severe complications, including neurological deficits, sepsis, and even mortality (10). In this context, metagenomic next-generation sequencing (mNGS) has emerged as a valuable complement to conventional culture-based methods, enabling non-targeted, rapid, and accurate identification of causative pathogens in brain abscess (11). This capability facilitates the prompt initiation of appropriate treatment, leading to improved outcomes and reduced risk of complications.

The updated guidelines on brain abscess diagnosis and management emphasize the critical role of anaerobic pathogens and advocate for the application of molecular techniques (12). However, the dearth of strong evidence from robust studies has hindered the widespread acceptance of these cutting-edge approaches. Addressing this knowledge gap, our study aimed to assess the diagnostic accuracy of mNGS in samples from brain abscess patients. The distinctive features of brain abscess, including the challenges associated with accessing purulent material and the low rate of positive blood cultures, significantly contribute to the complexity of diagnosis, underscoring the importance of our research.

Our study suggests that mNGS may potentially serve as a supplementary method in detecting pathogens, particularly anaerobes, in pus and cerebrospinal fluid (CSF) samples from brain abscess patients. These findings indicate that incorporating mNGS into diagnostic strategies may enhance our understanding and management of brain abscess, potentially leading to improved patient care and outcomes.

## MATERIALS AND METHODS

### Data collection

**Fig 1:**
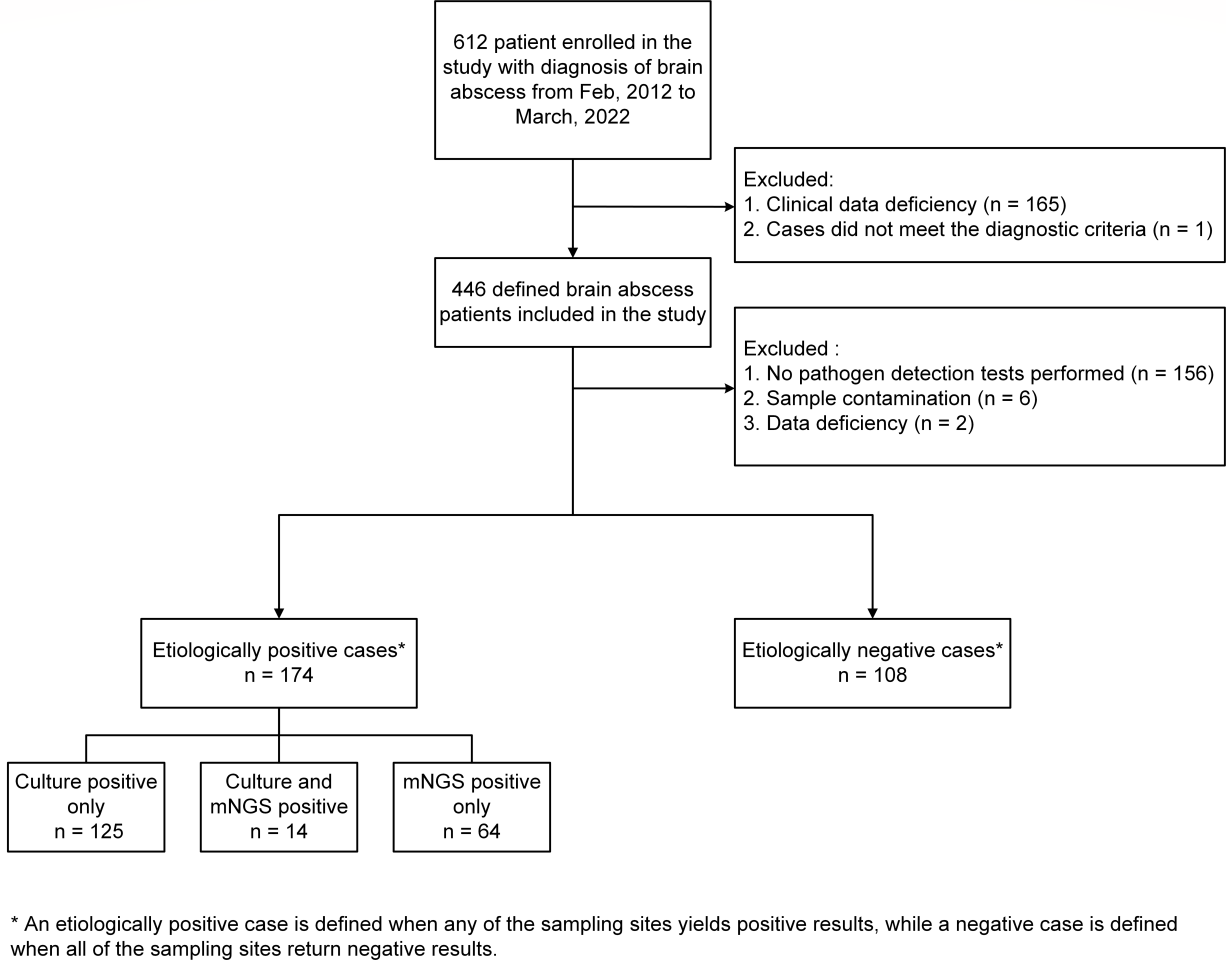
The workflow of the study.

We conducted a retrospective review of the electronic patient records from cases diagnosed with “brain abscess” or “cerebral abscess” at Huashan Hospital, affiliated with Fudan University, spanning from February 2012 to March 2022. Huashan Hospital is a national tertiary teaching hospital known for its expertise in neurology and infectious diseases. Patients who did not undergo pathogen detection tests, cases where sample contamination occurred during testing, or instances with insufficient data availability were excluded. The records of included patients were further reviewed to confirm clinical manifestations, laboratory findings, radiological reports, and microbiological results (Fig. 1).

### Radiological diagnosis

All patients underwent diagnostic cranial CT and/or MRI scans. Brain abscess cases were diagnosed based on the initial pre-treatment scans, with each scan independently reviewed and diagnosed by at least two radiologists. In high-risk patients, cardiac ultrasound and thoracoabdominal CT were conducted to identify brain abscess secondary to endocarditis, while abdominal ultrasound was utilized to detect infection dissemination leading to brain abscess.

### Microbiological diagnosis

Samples from blood, pus (from the brain or other organs), and CSF were processed in a central laboratory for pathogen identification. Given the dissemination patterns of brain abscesses and the challenges associated with obtaining brain parenchyma pus in internal medicine wards, we opted to include pus samples from non-brain organs in some of our patients. Patients included in this subgroup had documented dissemination routes and clinical courses consistent with brain abscess. Blood cultures were aseptically inoculated into BD BACTEC bottles and cultured on the Becton Dickinson BACTEC FX system. For the culture of pus and CSF, samples were inoculated into (i) cooked meat broth (extended incubation for 10 days in aerobic conditions) and (ii) blood, chocolate, and MacKonkey agar incubated in 5% CO_2_. The cultures were grown at 35–37℃, and the strains on the culture plate were further identified using matrix-assisted laser desorption/ionization time-of-flight mass spectrometry (MALDI-TOF MS).

For mNGS, samples from blood, pus (from the brain or other organs), and CSF were collected in 1.5 mL microcentrifuge tubes containing 1 g of 0.5 mm glass beads for DNA extraction. These tubes were attached to a horizontal platform on a vortex mixer and vigorously agitated at a speed of 2,800–3,200 rpm for 30 minutes. Subsequently, a 0.3 mL sample was transferred to a new microcentrifuge tube, and total DNA extraction was carried out using the TIANamp Micro DNA Kit (DP316, Tiangen Biotech, Beijing, China). A DNA library was constructed, involving DNA fragmentation (150 bp), end-repair, adapter ligation, and unbiased PCR amplification. The DNA libraries (200–300 bp) were subjected to quality control using the Agilent 2100 system. Libraries meeting the quality criteria were sequenced using the BGISEQ-200 platform.

After removing low-quality and short reads (<35 bp), the clean reads underwent alignment by the Burrows-Wheeler alignment tool. The alignment process first mapped the reads to the human reference databases, including hg19 and Yanhuang genome sequence. Then, the remaining reads were referred to the genome databases downloaded from the National Center for Biotechnology Information. The reference database consisted of 6,350 bacterial genomes, 4,945 viral taxa, 1,064 fungi, and 234 parasites associated with human infection.

The nucleic fragments annotated by mNGS are unable to differentiate between infection, colonization, or the residual fragments from treatment (13). Two independent infectious disease physicians assessed the patient’s condition, imaging findings, and treatment outcomes to evaluate whether the detected results of the patient samples were indicative of contamination. Viruses detected by mNGS were excluded, considering the typical pathogenesis of brain abscess involving bacteria, fungi, or parasites.

### Statistical methods

Data were collected and managed using Microsoft Excel, with all statistical analyses performed using GraphPad Prism 9 (GraphPad Soft-ware, Inc., La Jolla, CA, USA). The distribution of data was assessed by Shapiro–Wilk test. Comparisons between two groups were conducted using parametric *t*-tests for normally distributed data and non-parametric *t*-test for non-normally distributed data. Descriptive data were analyzed using the Chi-square test. *P* < 0.05 was considered statistically significant.

## RESULTS

### Patients’ characteristics

A total of 446 patients diagnosed with brain abscess were enrolled in the 10-year study. Among these patients, 69.5% were male and 30.5% were female, with a median age of 52 years. The most commonly reported symptoms were headache (58.3%) and fever (57.6%), while paralysis (20.9%) and epilepsy (17.0%) were less frequently observed. Among the underlying diseases, diabetes (11.7%) was the most frequently reported. CSF analyses demonstrated elevated leukocyte levels in 67.6% of patients, elevated protein levels in 88.4% of patients, and decreased glucose levels in 66.7% of patients. Disseminated infection was detected in 174 cases (39.0%) through cardia ultrasound and thoracoabdominal CT. Approximately half of the patients (52.0%) underwent surgical procedures, and the overall mortality rate during the study period was 10.1% during the study period. A summary of these findings is provided in Table 1.

**TABLE 1:** Patients characteristics

Characteristic	Value	Normal range
Female gender, *n* (%)	136 (30.5)	
Male gender, *n* (%)	310 (69.5)	
Median age, year (IQR)^*a*^	52 (39–64)	
Symptom, *n* (%)		
Headache	260 (58.3)	
Fever	257 (57.6)	
Paralysis	93/446 (20.9)	
Epilepsy	76/446 (17.0)	
Underlying disease		
Diabetes	52 (11.7)	
Autoimmune diseases	24 (5.4)	
Neoplasm	21 (4.7)	
Hematogenous	6 (1.4)	
Organ transplant	4 (0.9)	
Other chronic diseases Laboratory analysis, median (IQR)	14 (3.1)	
Leukocyte (10^9^ /L)	7.34 (5.25–10.13)	3.5–9.5
Neutrophil (%)	72.7 (61.8–81.8)	40–75
CRP (mg/L)	5.66 (3.03–22.01)	0–10
PCT (ng/L)	0.08 (0.04–0.185)	0–0.05
Cerebrospinal fluid, median (IQR)		
Leukocyte (10^6^ /L)	30 (5–230)	0–8
Protein (mg/L)	1,343 (729–2,533.5)	80–430
Glucose (mmol/L)	2.6 (2.0–3.1)	2.5–4.5
Disseminated infection (%)	174 (39.0)	
Surgery (%)	232 (52.0)	
Mortality (%)	45 (10.1)	

^*a*^ IQR, interquartile range; CRP, C-reactive protein; PCT, procalcitonin.

### Overall diagnostic performance among different microbiological methods

Samples from surgically obtained pus, CSF, and blood were subjected to conventional culture and/or mNGS. Of the 446 patients, 174 were identified as etiologically positive cases, while 108 were negative cases (Fig. 1). Blood cultures were performed in 139 patients, CSF cultures in 128 patients, pus cultures in 138 patients, and mNGS tests in 74 patients. All mNGS samples included in our study passed quality control.

A total of 275 pathogens were detected, comprising 249 bacteria, 20 fungi, 5 mycobacteria, and 1 parasite (Fig. S1). Gram-negative bacteria (35.6%) and gram-positive bacteria (54.9%) accounted for the majority of the pathogen spectrum. The most frequently detected gram-negative bacteria were *Klebsiella* spp. (*n* = 26) and *Fusobacberium* spp. (*n* = 14), while *Streptococcus* spp. (*n* = 83) and *Staphylococcus* spp. (*n* = 24) were the most common gram-positive bacteria identified in the study.

### mNGS is more efficient than conventional cultures in diagnosing brain abscess

A total of 38 isolates were reported from blood cultures among the brain abscess patients (Table 2). The most prevalent pathogens were *Streptococcus* spp. (*n* = 12, 31.6%), followed by *Staphylococcus* spp. (*n* = 10, 26.3%) and *Klebsiella* spp. (*n* = 6, 15.8%). *Candida* spp. accounted for the fungal isolate (*n* = 3, 7.9%).

**TABLE 2:** Isolates from blood culture among brain abscess patients

Classification	Pathogen (genus)	Isolate (%)	Pathogen (species)	Isolate
Gram positive	*Streptococcus* spp.	12 (31.6)	*Streptococcus palliative*	3
			*Streptococcus oralis*	3
			*Streptococcus pneumoniae*	2
			*Streptococcus grazi*	1
			*Streptococcus vialiensis*	1
			*Streptococcus constellatus*	1
			*Streptococcus mutants*	1
	*Staphylococcus* spp.	10 (26.3)	*Staphylococcus aureus*	5
			*Staphylococcus cephalicus*	2
			*Staphylococcus epidermididis*	1
			*Staphylococcus caprae*	1
	*Listeria* spp.	1 (2.6)	*Listeria monocytogenes*	1
	*Enterococcus* spp.	1 (2.6)	*Enterococcus faecalis*	1
	*Nocardia* spp.	1 (2.6)	*Nocardia derma*	1
Gram negative	*Klebsiella* spp.	6 (15.8)	*Klebsiella pneumoniae*	6
	*Pseudomonas* spp.	2 (5.3)	*Pseudomonas aeruginosa*	1
			*Pseudomonas Fluorescens*	1
	*Enterobacteriaceae* spp.	1 (2.6)	*Enterobacter cloacae*	1
	*Brevundimonas* spp.	1 (2.6)	*Brevundimonas diminuta*	1
Fungus	*Candida* spp.	3 (7.9)	*Candida albicans*	2
			*Candida parapsilosis*	1
Total		38 (100)		37^*a*^

^*a*^ Due to one blood sample yielding only *Staphylococcus* genus in the culture report, the number of species is one less than the genus.

**Fig 2:**
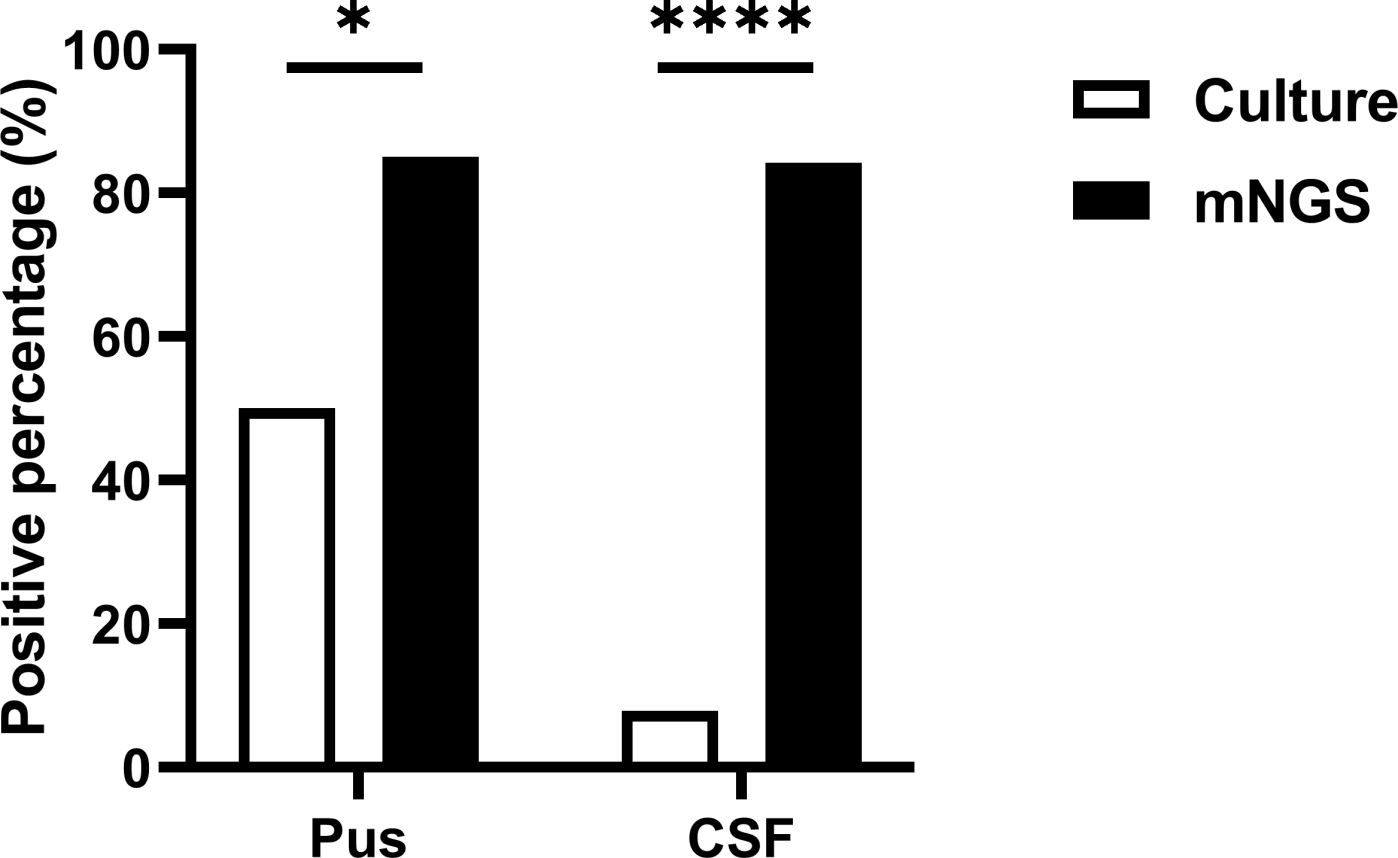
The overall positive percentage of pathogens between culture and mNGS in CSF and pus. *P < 0.05; ****P < 0.0001.

Concurrently, cultures and mNGS tests were performed on pus samples from 20 patients and CSF samples from 38 patients, which were included for further analysis. mNGS demonstrated significantly higher overall pathogen positive rates in pus samples (85.0% vs 50.0%, *P* = 0.0181) and CSF samples (84.2% vs 7.9%, *P* < 0.0001) compared to culture (Fig. 2). Among the 128 CSF cultures analyzed, no instances of polymicrobial infection were observed. Among the 138 cases analyzed in pus cultures, 2 cases displayed mixed infections of aerobic and anaerobic bacteria. However, mNGS analysis identified mixed infections of aerobic and anaerobic bacteria in 15 out of the 74 cases examined. These findings indicate the effectiveness of mNGS in diagnosing a broader spectrum of pathogens in brain abscesses compared to conventional cultures.

### mNGS reveals a higher detection rate of anaerobes in brain abscess

**Fig 3:**
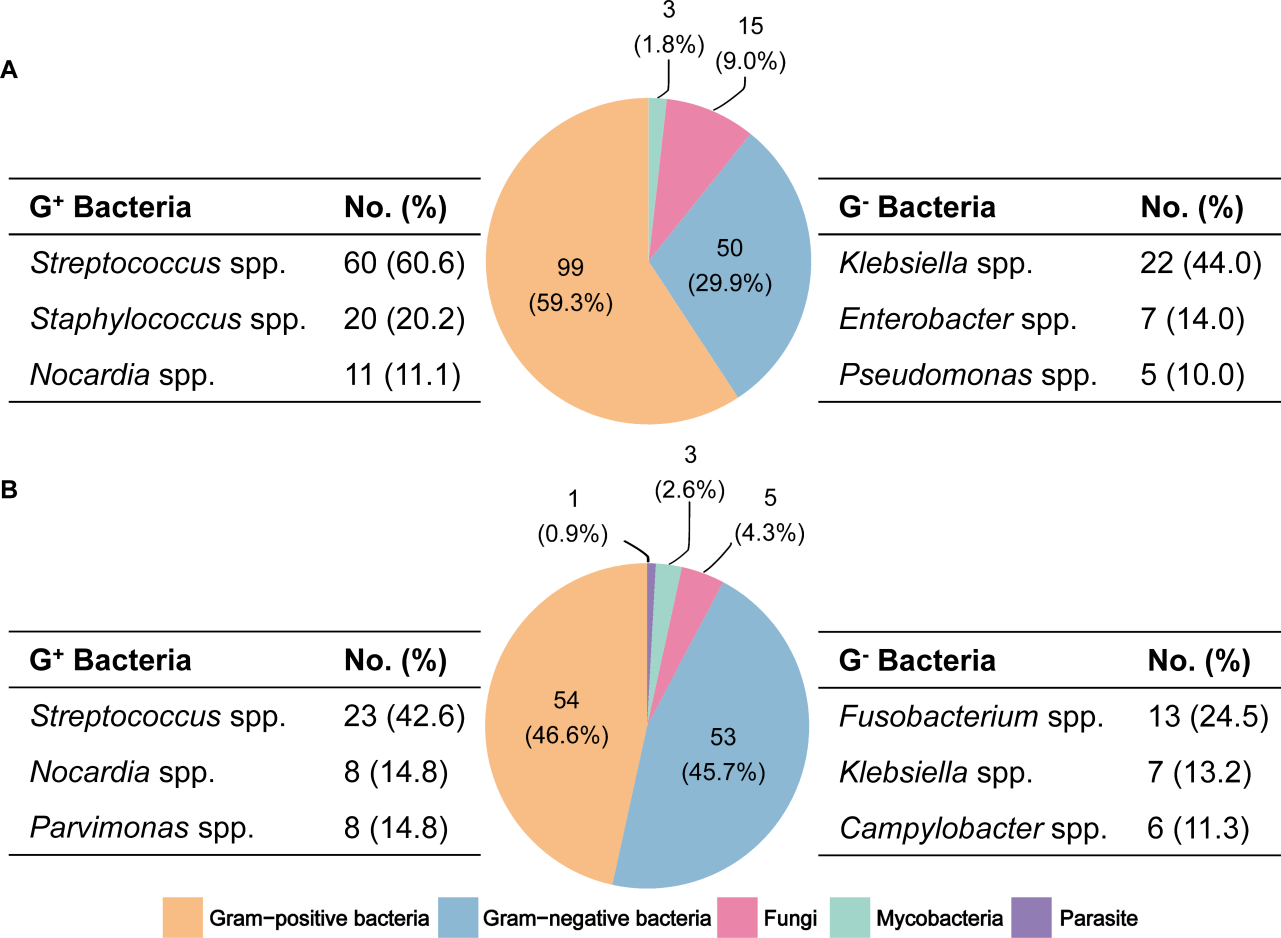
The pathogen spectrums identified by conventional culture (**A**) and mNGS (**B**).

The culture and mNGS tests revealed pathogen spectrums with different ratios of gram-positive and gram-negative bacteria (Fig. 3). Culture tests revealed 167 results. Gram-positive bacteria accounted for 59.3% (*n* = 99) of the identified pathogens, within which *Streptococcus* spp. (*n* = 60, 60.6%), *Staphylococcus* spp. (*n* = 20, 20.2%), and *Nocardia* spp. (*n* = 11, 11.1%) were most frequently detected. Gram-negative bacteria accounted for 29.9% (*n* = 50), where *Klebsiella* spp. (*n* = 22, 44.0%), *Enterobacter* spp. (*n* = 7, 14.0%), and *Pseudomonas* spp. (*n* = 5, 10.0%) being the most commonly detected. On the other hand, mNGS tests reported 116 pathogens. Gram-positive bacteria accounted for 46.6% (*n* = 54) of the identified pathogens, with *Streptococcus* spp. (*n* = 23, 42.6%), *Nocardia* spp. (*n* = 8, 14.8%), *Parvimonas* spp. (*n* = 8, 14.8%), and others included. Gram-negative bacteria accounted for 45.7% (*n* = 53) of the identified pathogens, including *Fusobacterium* spp. (*n* = 13, 24.5%), *Klebsiella* spp. (*n* = 7, 13.2%), *Campylobacter* spp. (*n* = 6, 11.3%), and others. The detection rate of mycobacteria was similar in both culture and sequencing methods, while the positive rate of fungi was higher in culture than mNGS methods.

We also performed a parallel analysis of pathogen identification results from different methods, including pus/CSF culture, blood culture, and mNGS (Table S1). In culture-negative cases, mNGS identified 43 distinctive taxa, including a significant number of rare pathogens and fastidious bacteria. Anaerobes such as *Fusobacterium* spp., *Parvimonas* spp., *Porphyromonas* spp., and *Prevotella* spp. were detected exclusively by mNGS. The discrepancy in pathogen detection, particularly driven by anaerobes, highlights the ability of mNGS to improve the detection rate of anaerobes in brain abscess compared to conventional culture methods.

### mNGS improves the detection rate of anaerobes in CSF to diagnose brain abscess

**Fig 4:**
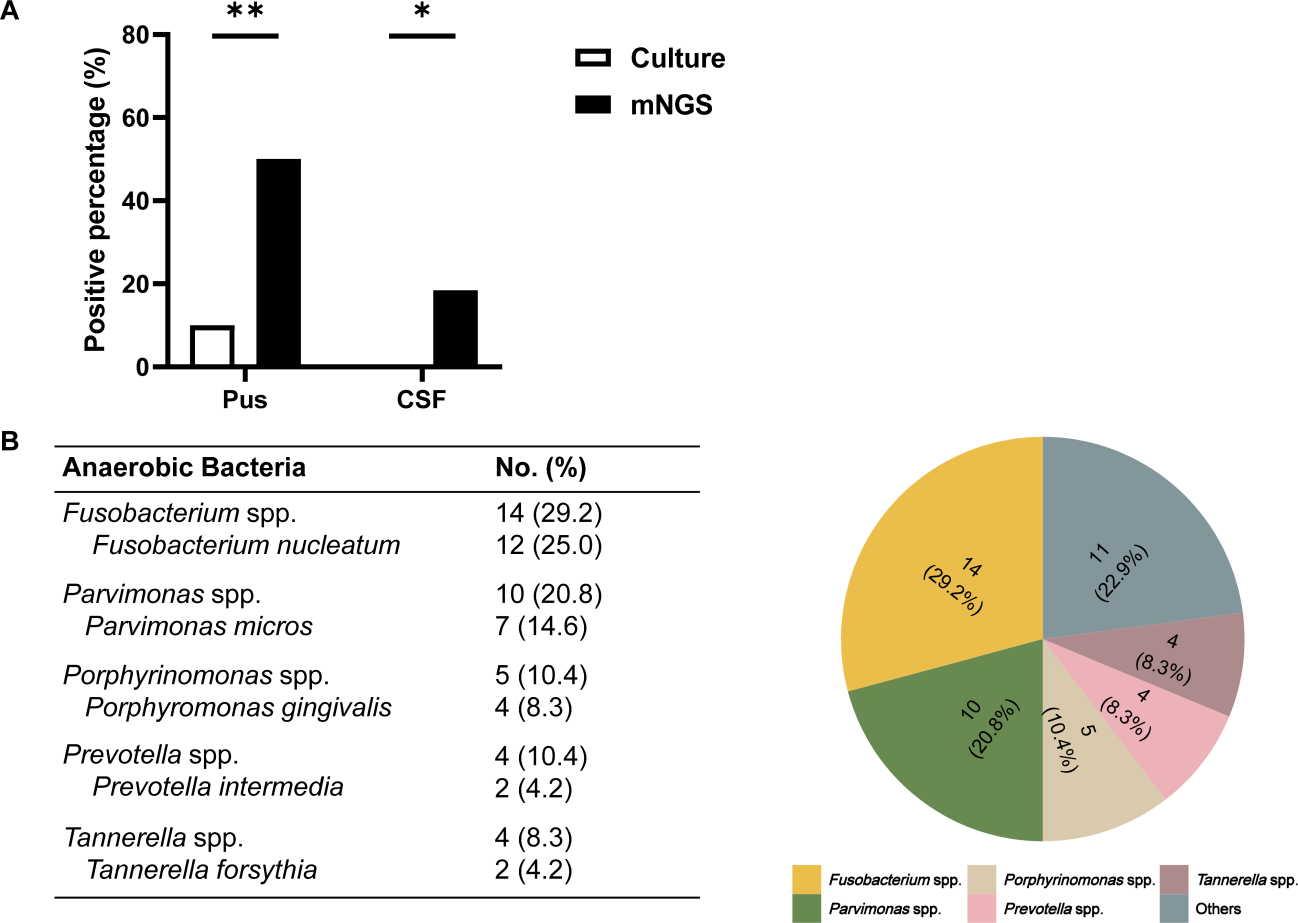
The positive percentage of anaerobes in brain abscess patients. (**A**) The positive percentage of anaerobes between culture and mNGS in pus and CSF specimen. *P < 0.05; **P < 0.01. (**B**) Distribution (pie chart) and the most frequent (table) anaerobic bacteria in the brain abscess patients.

Comparatively, mNGS demonstrated a significantly higher detection rate for anaerobes, with notably elevated positive detections in both pus samples (50.0% vs 10%, *P* = 0.0058) and CSF samples (18.4% vs 0%, *P* = 0.0115, Fig. 4A). Notably, the positive detection rate for anaerobes in pus samples was lower than the overall pathogen detection rate (Fig. 2). These findings suggest the potential of mNGS as a valuable tool for diagnosing anaerobe-caused brain abscesses in CSF specimens. The spectrum of identified anaerobes from both microbiological methods yielded 48 anaerobic isolates, while 39 (81.3%) isolated were detected by mNGS. The most frequent isolates included *Fusobacterium* spp. (*n* = 14, 29.2%), *Parvimonas* spp. (*n* = 10, 20.8%), *Porphyrinomonas* spp. (*n* = 5, 10.4%), *Prevotella* spp. (*n* = 4, 10.4%), and *Tannerella* spp. (*n* = 4, 8.3%). Among these, *Fusobacterium* spp. was the most frequently detected anaerobe in patients with intracranial abscess, comprising nearly a third of all anaerobes (Fig. 4B). These results indicate the significance of mNGS in diagnosing anaerobic infections in brain abscess, particularly in CSF specimens.

## DISCUSSION

Brain abscess represents a serious clinical condition associated with long-term neurological morbidity and mortality (2, 10, 14). Despite advancement in cranial radiology and antimioicrobial therapy, diagnosing brain abscess remains challenging due to a variety of factors, including the difficulty in accurately identifying underlying pathogens and the potential for polymicrobial infections involving anaerobic bacteria (14, 15). To address these challenges, our study collected data over a 10-year period from brain abscess patients at Huashan hospital, a tertiary referral center, using advanced molecular techniques such as mNGS and MALDI-TOF MS system (16) to assist pathogen detection. By comparing the effectiveness of different detection methods, our study provides valuable evidence that mNGS has the potential to optimize the clinical management of brain abscess.

In previous studies, the most common causative microorganisms in brain abscess were *Streptococcus* spp., *Staphylococcus* spp., enteric gram-negative bacteria, *Fusobacteirum* spp., and *Aggregatibacter* spp. (2, 6, 12, 17). Our study corroborated the predominance of *Streptococcus* and *Staphylococcus* species. However, the utilization of mNGS expanded the pathogen landscape, raising the prevalence of gram-negative bacteria (45.7%) in brain abscess cases. Specifically, the detection of gram-negative anaerobes, particularly *F. nucleatum*, contributed to a higher proportion of gram-negative bacteria. Fusobacteria are anaerobic bacilli commonly found in various ecological niches, including the oral cavity, gastrointestinal tract, and female genital tract (18). Despite their significance, anaerobes are often overlooked due to limitations in conventional techniques, and few studies have focused on brain abscess caused by anaerobic pathogens (19–22).

The low detection rate of anaerobic bacteria in abscess foci can be attributed to several factors. Traditional microbiological techniques primarily optimized for aerobic or facultative anaerobic organisms may result in poor growth of anaerobes or overgrowth of faster-growing aerobic bacteria (23). Additionally, anaerobic bacteria have specific requirements for sampling, transportation, and extended incubation times compared to aerobic bacteria. Failure to meet these requirements can compromise the recovery of anaerobes (24). A systemic review was conducted on studies published between 1998 and 2022 to investigate brain abscess caused by anaerobic bacteria. This review included 28 studies encompassing 6,167 patients (25). Among these cases, 715 (11.5%) were attributed to anaerobic bacteria, with *Fusobacterium* spp. accounting for 12.4% of the isolates. Our study underscores the effectiveness of molecular techniques, specifically mNGS, in augmenting the detection rate of anaerobes.

mNGS is a hypothesis-free high-throughput technique renowned for its ability to comprehensively characterize the microbial population present in clinical specimens (26). This approach offers significant advantages over traditional culture methods, providing a definitive etiologic diagnosis and valuable antimicrobial susceptibility information, particularly for fastidious bacteria (11, 27, 28). In comparison to Sanger and 16S rRNA sequencing methods, mNGS surpasses them by offering higher taxonomic resolution, enabling more comprehensive analyses of microbial functions, and the ability to detect novel or poorly characterized organisms (23, 29).

**Fig 5:**
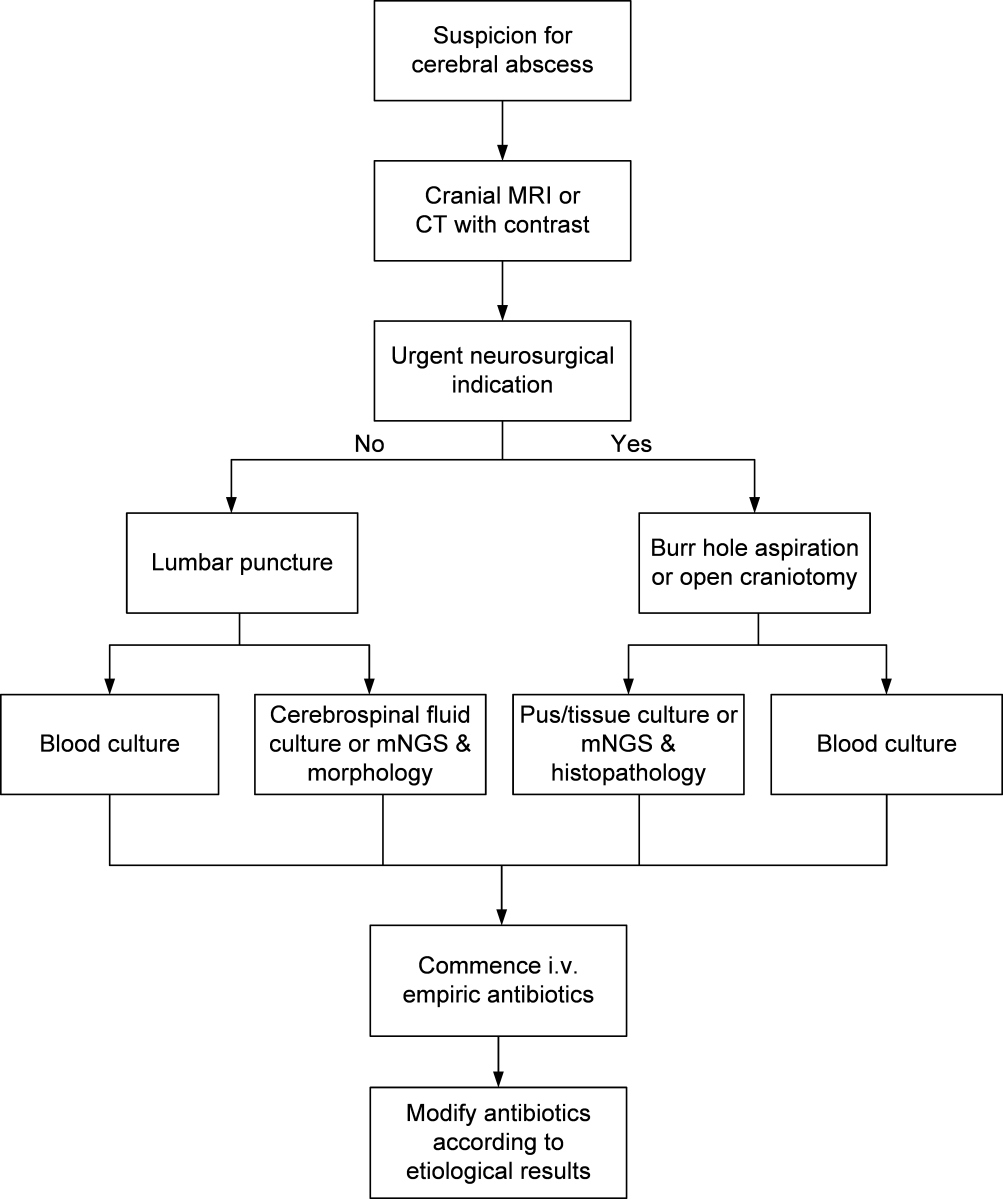
The optimized diagnostic strategy of brain abscess proposed benefit from mNGS.

Our study also investigated the effectiveness of different specimen types for the etiological diagnosis of brain abscess, revealing that pus specimens exhibited the highest pathogen detection rate, followed closely by CSF specimens. Blood specimens yielded the lowest detection rate, likely due to the localized nature of pathogens within the brain abscess, which may not be present in the peripheral blood. This disparity in detection rates between specimen types is noteworthy, especially when compared to other anatomical sites like liver abscess, where the blood culture positive rate for brain abscess remains relatively low (2, 30). Although, the majority of existing research on brain abscesses heavily relies on pus samples, with limited reports on CSF microbiology (12, 14, 31). Our study unveiled that mNGS demonstrated comparable rates of pathogen detection for both pus and CSF specimens. This finding suggests the viability of CSF specimens as a potential alternative when neurosurgery access is challenging. The prior diagnostic strategy for brain abscess has focused on blood and pus cultures, overlooking the value of CSF. We propose an updated diagnostic and therapeutic strategy according to our findings (Fig. 5).

Several studies have examined the performance of molecular techniques for diagnosing brain abscess. However, these studies have limitations as they primarily rely on 16S rRNA-based sequencing of pus materials (23, 29, 32). In clinical practice, many patients with brain abscess fail to meet the criteria for surgery or are unable to obtain pus specimens due to technical limitations, severe illness, or other uncontrollable factors. In such cases, CSF represents a potential avenue for diagnosing brain abscess. While recent case reports have highlighted the potential of mNGS in diagnosing brain abscess through CSF analysis, comprehensive research encompassing large sample sizes to evaluate the performance of mNGS is still limited (33–35). Consequently, our study aims to address this gap by providing robust evidence of the superiority of mNGS over conventional culture methods, particularly in detecting anaerobes in CSF samples. Molecular biology techniques can identify genetic fragments from both viable and non-viable bacteria, enabling accurate detection even in the presence of antibiotics. This attribute is crucial, considering the challenges associated with sampling in brain abscess patients. Moreover, a previous study conducted in our institution demonstrated that mNGS has been developed to provide results in less than 48 hours, which substantially expedites the diagnosis of brain abscess (36).

The study has some inherent limitations that should be acknowledged. First, as a single-center study, the generalizability of the findings may be limited due to the restricted sample size from a specific region and population. Second, being a retrospective study, there is a possibility of bias in data collection and recording, which may impact the accuracy of the results. Lastly, while mNGS demonstrated an improved pathogen detection rate, the study did not establish a direct relationship between this improvement and enhanced patient treatment outcomes.

In conclusion, our study provides updated insights into the pathogen spectrum and clinical characteristics of brain abscess patients. Our findings suggest that anaerobic infections are more common than previously thought. Apart from the traditional methods, mNGS can assist to detect more anaerobes for brain abscess. We also found that pus specimens are the most effective for microbiological diagnosis, followed by CSF specimens, and that blood specimens have the lowest detection rate. In situations where pus specimens are difficult to obtain, CSF specimen can serve as a reference.

## Data Availability

All the data supporting the findings are provided in the article. Any additional data are available on request to the corresponding authors.

## SUPPLEMENTAL MATERIAL

Supplemental material spectrum.00325-24-s0001.docx Fig. S1; Table S1.

ASM does not own the copyrights to Supplemental Material that may be linked to, or accessed through, an article. The authors have granted ASM a non-exclusive, world-wide license to publish the Supplemental Material files. Please contact the corresponding author directly for reuse.
