# Supplementary material for: Augmented pathogen detection in brain abscess using metagenomic next-generation sequencing: a retrospective cohort study
Source: Microbiol Spectr. 2024 Sep 12;12(10):e00325-24. doi: 10.1128/spectrum.00325-24 (PMC11448231; doi:10.1128/spectrum.00325-24)
Supplement: Supplemental material — Fig. S1; Table S1. [file spectrum.00325-24-s0001.docx]

**Figure S1.** The overall pathogen spectrum of the brain abscess patients in our study.


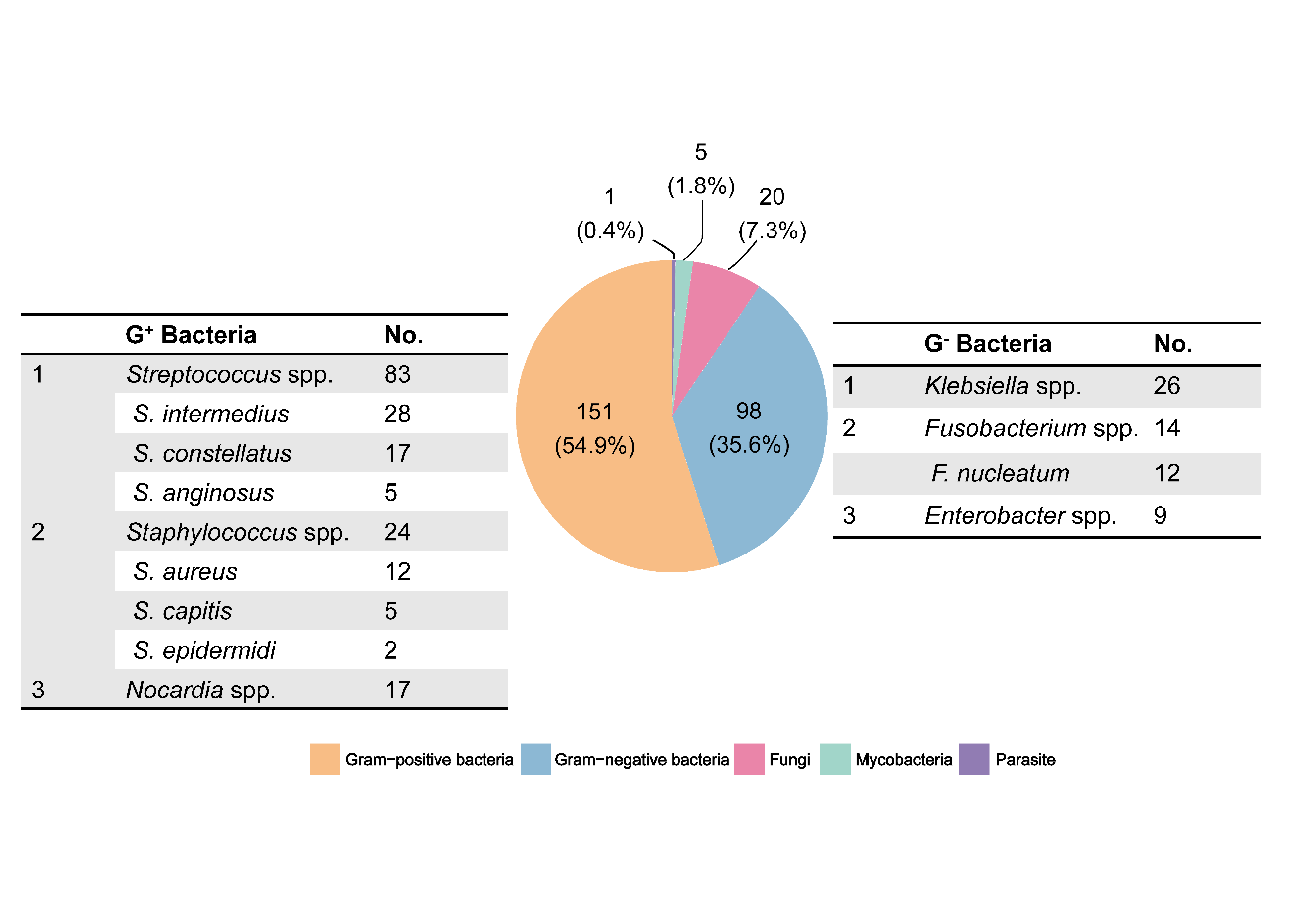


**Table S1.** The discrepancy between culture and mNGS methods.

| **Discrepancy between methods** | **Difference in the identification of species or genera (No.)** |
| --- | --- |
| pus/CSF culture positive but mNGS negative | None |
| pus/CSF culture positive but blood culture negative | *Streptococcus* spp. (12), *Staphylococcus* spp. (2), *Nocardia* spp. (1)  *Actinomyces* spp. (1)*  *Klebsiella* spp. (7), *Acinetobacter* spp. (2), *Escherichia* spp. (1), *Pseudomonas* spp*.* (1),  *Fusobacterium* spp. (1), *Bacteroides* spp. (1)  *Mycobacteria* spp. (2)  *Aspergillus* spp. (1), *Candida* spp. (1), *Cryptococcus* spp. (1) |
| blood culture positive but mNGS negative | None |
| blood culture positive but pus/CSF negative | *Staphylococcus* spp. (1), *Nocardia* spp. (1)  *Klebsiella* spp. (1), *Brevundimonas* spp. (1) |
| mNGS positive but pus/CSF culture negative | *Streptococcus* spp. (22), *Staphylococcus* spp. (3), *Nocardia* spp. (3), *Listeria* spp. (2), *Corynebacterium* spp. (2), *Granulicatella* spp. (1),  *Parvimonas* spp. (7), *Filifactor* spp. (1)  *Klebsiella* spp. (6), *Campylobacter* spp. (6), *Aggregatibacter* spp. (3), *Pseudomonas* spp. (2), *Shigella* spp. (2), *Escherichia* spp. (2), *Eikenella* spp. (1), *Spirochaeta* spp. (1)  *Fusobacterium* spp. (16), *Porphyromonas* spp. (3), *Prevotella* spp. (3), *Tannerella* spp. (2)  *Aspergillus* spp. (3), *Candida* spp. (2) |
| mNGS positive but blood culture negative | *Streptococcus* spp. (16), *Staphylococcus* spp. (2), *Nocardia* spp. (2), *Listeria* spp. (1), *Enterococcus* spp. (1), *Granulicatella* spp. (1), *Corynebacterium* spp. (1),  *Parvimonas* spp. (6), *Finegoldia* spp. (1),  *Klebsiella* spp. (5), *Campylobacter* spp. (4), *Shigella* spp. (3), *Escherichia* spp. (2), *Pseudomonas* spp. (1), *Aggregatibacter* spp. (1),  *Eikenella* spp. (1)*, Spirochaeta* spp. (1), *Citrobacter* spp. (1)  *Fusobacterium* spp. (15), *Prevotella* spp. (3), *Porphyromonas* spp. (2), *Tannerella* spp. (2),  *Mycobacterium* spp. (2),  *Aspergillus* spp. (2), *Cryptococcus* spp. (1), *Candida* spp. (2),  *Baboon Balamshi Amoeba* (1) |

* Anaerobic bacteria are highlighted with underlines.
